# Protein oxidation and peroxidation

**DOI:** 10.1042/BJ20151227

**Published:** 2016-03-29

**Authors:** Michael J. Davies

**Affiliations:** * Department of Biomedical Sciences, Panum Institute, University of Copenhagen, Blegdamsvej 3, Copenhagen 2200, Denmark

**Keywords:** amino acid oxidation, hydroperoxides, peroxidation, peroxides, protein oxidation, radicals, singlet oxygen, UV

## Abstract

Proteins are major targets for radicals and two-electron oxidants in biological systems due to their abundance and high rate constants for reaction. With highly reactive radicals damage occurs at multiple side-chain and backbone sites. Less reactive species show greater selectivity with regard to the residues targeted and their spatial location. Modification can result in increased side-chain hydrophilicity, side-chain and backbone fragmentation, aggregation via covalent cross-linking or hydrophobic interactions, protein unfolding and altered conformation, altered interactions with biological partners and modified turnover. In the presence of O_2_, high yields of peroxyl radicals and peroxides (protein peroxidation) are formed; the latter account for up to 70% of the initial oxidant flux. Protein peroxides can oxidize both proteins and other targets. One-electron reduction results in additional radicals and chain reactions with alcohols and carbonyls as major products; the latter are commonly used markers of protein damage. Direct oxidation of cysteine (and less commonly) methionine residues is a major reaction; this is typically faster than with H_2_O_2_, and results in altered protein activity and function. Unlike H_2_O_2_, which is rapidly removed by protective enzymes, protein peroxides are only slowly removed, and catabolism is a major fate. Although turnover of modified proteins by proteasomal and lysosomal enzymes, and other proteases (e.g. mitochondrial Lon), can be efficient, protein hydroperoxides inhibit these pathways and this may contribute to the accumulation of modified proteins in cells. Available evidence supports an association between protein oxidation and multiple human pathologies, but whether this link is causal remains to be established.

## INTRODUCTION

Biological systems are continually exposed to endogenous and exogenous oxidants (both free radicals–species with an unpaired electron–and two-electron oxidants). The processes that give rise to these oxidants will not be covered further here in detail as they have been reviewed extensively (reviewed [1]), though a brief list is given in Table 1. Under normal circumstances the formation and reactions of these species are limited by defensive systems within cells and organisms. These include low-molecular-mass scavengers (e.g. ascorbic acid, thiols, quinols, tocopherols, carotenoids, polyphenols, urate), enzymes that remove either oxidants directly (e.g. superoxide dismutases) or their precursors (e.g. peroxiredoxins, glutathione peroxidases and catalases that remove peroxides), and enzyme systems that repair damage (methionine sulfoxide reductases, disulfide reductases/isomerases, sulfiredoxins) or remove damaged material (e.g. proteasomes, lysosomes, proteases, phospholipases, DNA repair enzymes).

**Table 1:** Examples of endogenous and exogenous factors that result in oxidant formation

Endogenous	Exogenous
Electron transport chains (mitochondria, endoplasmic reticulum, plasma membrane)	Radiation (high energy, UV, visible light + sensitizer, thermal, ultrasound)
Haem protein/enzyme reactions (e.g. haemoglobin, myoglobin, cytochromes such as cytochrome P_450_)	Metabolism of chlorinated hydrocarbons, drugs, nitro compounds, paracetamol, ethanol
Peroxidases	Nitrogen oxides (NO*_x_*)
Nitric oxide synthases (NOS)	Particulates (e.g. diesel particles)
NADPH oxidases (NOx)	Mineral fibres and dusts (e.g. asbestos)
Xanthine oxidase	Ozone
Lipoxygenases	Sulfur oxides (SO*_x_*)
Prostaglandin synthases	Oxidized foodstuffs
Autooxidation of glucose, thiols, catecholamines, metal ions	Combustion processes (e.g. smoking)Metal ion overload (e.g. Fe, Cu)

Despite this diversity, elevated levels of oxidative damage have been detected in a wide range of human, animal, microbial and plant systems (reviewed [1]). This may be due to increased oxidant formation or exposure, a decrease or failure of defence systems, or both. In some cases it is clear why this imbalance arises (e.g. exposure to high energy radiation, genetic faults that result in decreased levels or absence of repair enzymes), but in many cases both factors are likely to be important, as many defensive systems are themselves subject to alteration in level and or activity (e.g. as a result of alterations in transcription and/or translation, or direct damage), or have a requirement for cofactors that can be readily depleted/oxidized. It is widely reported that aging results in an overall decline in the activity of many enzymes, and lower levels of many essential trace elements and metabolites, and this decline can be readily accelerated by disease or external factors (reviewed [1]). Much of these data are associative, and there are only a limited number of cases where causality has been established; it is likely that most examples of increased oxidative damage arise from a conjunction of multiple effects with this varying from subject to subject.

A wide range of different oxidants (both radical and two-electron) can be generated *in vivo*, with these arising from multiple external and endogenous processes. These species vary markedly in their reactivity and the resulting damage is highly variable and complex. Some highly reactive species, such as hydroxyl radicals (HO^•^, which can arise from exposure to high energy radiation and metal ion-catalysed decomposition of hydrogen peroxide, H_2_O_2_) are capable of oxidizing nearly all biological targets, with second order rate constants near the diffusion limit (i.e. *k* ∼ 10^9^–10^10^ M^−1^·s^−1^; Table 2). Due of the abundance of targets *in vivo*, this results in a microsecond lifetime, and very limited diffusion from its site of generation, so most HO^•^-induced damage is site specific (e.g. at sites of metal ion binding, or within highly focused radiation beams).

**Table 2:** Selected apparent second order rate constants for reaction of HO^•^ with biological macromolecules and antioxidants Data from [28].

Target	Apparent second order rate constant (M^−1^·s^−1^)
DNA	8×10^8^
RNA	1×10^9^
Hyaluronan	7×10^8^
Linoleic acid	9×10^9^
Collagen	4×10^11^
Albumin	8×10^10^
Ascorbate	1×10^10^
GSH	1.4×10^10^
Trolox C (water-soluble analogue of α-tocopherol)	6.9×10^9^

**Figure 1:**
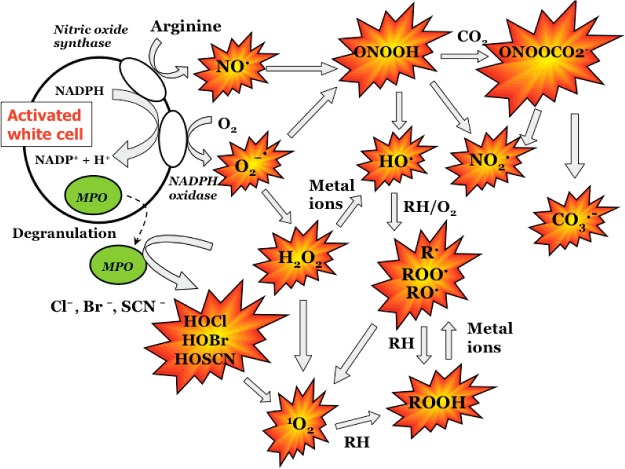
Examples of oxidant species (both two-electron oxidants and radicals) generated from activated leucocytes and their interconversion Abbreviation: MPO, myeloperoxidase.

Although HO^•^ is highly reactive and short-lived, other radicals are so long-lived that they can be isolated and purchased from commercial sources. Less chemically reactive species have longer biological half-lives and can diffuse long distances *in vivo*, though this can be limited by physical barriers, charge interactions and hydrophobicity/hydrophilicity. Such diffusion can result in remote effects making determination of the site and mechanism of radical formation complex. Furthermore it is well established that many of these species can interconvert and give rise to secondary oxidants, of different reactivity and lifetimes than the initial species (Figure 1). Thus determination of the site of oxidant formation, and the contribution of different oxidants to the overall extent of damage detected *in vivo* is challenging. Understanding the nature and reactivity of potential oxidants, and the patterns and extents of damage that they induce is therefore critical.

Approximate diffusion radii have been calculated for some biological oxidants with these values ranging from a few nm for HO^•^ to 1.5 mm for H_2_O_2_ (cf. typical cell diameters of 20 μm) [2,3]. These data need to be treated with care, as they are markedly dependent on the input data in the calculations, such as target concentrations, as well as other factors such as electronic effects, hydrophobicity/hydrophilicity, viscosity and temperature. This variation is exemplified by the ∼10-fold difference calculated for the powerful oxidant peroxynitrous acid in different biological milieus (0.5, 3 and 5.5 μm in erythrocytes, mitochondria and plasma respectively [3]), with this variation arising primarily from differences in the concentration of free and protein-bound Cys residues which are major targets for this oxidant.

The rate constants for reaction of different oxidants with a fixed concentration of a single biological target can vary by >10^10^. As an example, Table 3 provides apparent second order rate constants, *k*, for some common biologically relevant oxidants with the (free) amino acid methionine (Met). There can also be enormous variations in the rate constants for a single oxidant with different biological targets. Thus in contrast with HO^•^ where there are only relatively minor variations in *k* between targets (see above), other oxidants such as hypochlorous acid (HOCl, a major oxidant generated at sites of inflammation by neutrophils and monocytes) react with the various side chains present on proteins with *k* values that vary by ∼10^11^ (Table 4). Reactivity is also critically dependent on the environment of the oxidant and target; this is illustrated in Table 5, which provides rate constants for a number of oxidants with the same amino acid (Cys) in different environments–from free amino acid to the active site of specific enzymes. These data vary by ∼10^8^ as a result of environmental and structural factors that make some Cys residues particularly reactive (e.g. in peroxiredoxins) compared with other proteins, the Cys-containing tripeptide glutathione GSH, and the free amino acid [4–6].

**Table 3:** Selected apparent second order rate constants for reaction of some biological oxidants with the free amino acid methionine Data from [7,28].

Reactant	Apparent second order rate constant (M^−1^·s^−1^)
HO^•^	7×10^9^
CO_3_^−^•^^	1.2×10^8^
HOCl	3.8×10^7^
Singlet oxygen (^1^O_2_)	2×10^7^
Ozone (O_3_)	5×10^6^
CF_3_CHClOO^•^	1.4×10^6^
N_3_^•^	<10^6^
ONOO^−^/ONOOH	3.6×10^2^
O_2_^−^•^^	<0.3
H_2_O_2_	2×10^−2^
NO^•^	Very slow

**Table 4:** Selected apparent second order rate constants for reaction of HOCl with amino acid side chains, backbone amides and models of these structures Data from [278,281,282].

Side chain (model compound examined)	Apparent second order rate constant (M^−1^·s^−1^)
Cysteine side chain (cysteine)	3.6×10^8^
Glutathione (GSH)	1.2×10^8^
Methionine side chain (*N*-acetyl-Met-OMe)	3.4×10^7^
Cystine disulfide (3,3′-dithiodipropionic acid)	1.6×10^5^
Histidine side chain (4-imidazoleacetic acid)	1.2×10^5^
α-Amino group (Gly)	1.0×10^5^
Lysine side-chain amine (*N*-α-acetyl-Lys)	7.9×10^3^
Tryptophan side chain (*N*-acetyl-Trp)	7.8×10^3^
Tyrosine (*N*-acetyl-Tyr)	47
Arginine side chain (ethyl guanidine)	19
Amide bond [Cyclo-(Gly)_2_]	25
Amide bond [Cyclo-(Ala)_2_]	8.2
Glutamine/asparagine (propionamide)	0.041

**Table 5:** Apparent second order rate constants for reaction of selected biological oxidants with the amino acid cysteine in different environments at neutral pH (∼7.4) and ∼22°C Data from [4-6,47,275-282].

Oxidant	Cysteine environment	Apparent second order rate constant (M^−1^·s^−1^)
HOCl	Free amino acid	3.6×10^8^
	Cys in GSH	1.2×10^8^
HOSCN	Free amino acid	7.8×10^4^
	Cys in GSH	2.5×10^4^
	Cys-34 in BSA	7.6×10^4^
ONOOH	Free amino acid	2.6×10^3^
	Cys in GSH	7.3×10^2^
	Cys-34 in BSA	3.8×10^3^
	Active site Cys in peroxiredoxin 5	7×10^7^
H_2_O_2_	Free amino acid	0.84
	Cys in GSH	0.42
	Cys-34 in BSA	2.3
	Active site Cys in peroxiredoxin 2	∼10^7^

## PROTEINS ARE MAJOR TARGETS FOR OXIDATION

The extent of damage to biological targets depends on a range of factors including:

(1) the concentration of particular targets,
(2) the rate constant for reaction of oxidant with target
(3) the location of the target relative to that of the oxidant
(4) the occurrence of secondary damaging events, including chain reactions
(5) intra- and inter-molecular transfer reactions, and
(6) the possibility and extent of repair and oxidant scavenging reactions

**Figure 2:**
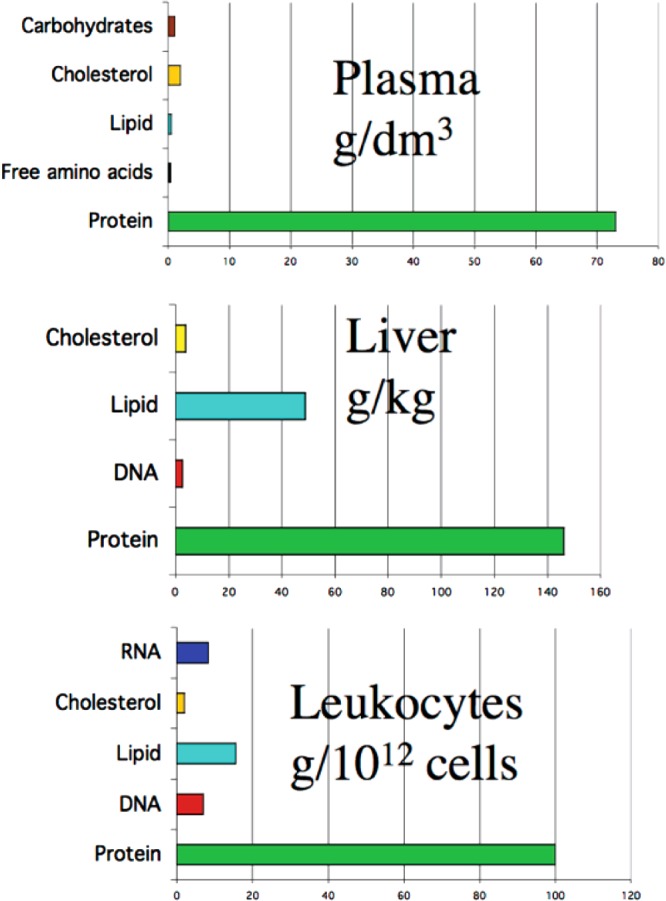
Abundance of potential targets for one- and two-electron oxidants in various biological systems, including plasma, liver and leucocytes Replotted data from [7]: Davies, M.J. (2005) The oxidative environment and protein damage. Biochim. Biophys. Acta 1703, 93–109.

The relative contributions of these different factors cannot be easily generalized, or ranked in terms of importance, however the first two are clearly of critical importance. As proteins are the major (non-water) components of most biological systems (Figure 2) with concentrations in plasma of 1–3 mM, and 5–10 mM in cells (calculated assuming an average protein molecular mass of 25–50 kDa) these are likely to be major targets [7]. Combination of these data with rate constant data allows calculations to be made as to the fate of oxidants. Thus for leucocytes ∼69% of HO^•^ generated by γ-radiation (a “clean” source of this radical), are thought to react with proteins [8], and a similar number has been obtained for the first excited state of molecular oxygen (singlet oxygen, ^1^O_2_) [9] and other oxidants. These data are crude extrapolations as they assume reaction in homogeneous solution and with unencumbered access, which is far from biological reality, but these data do provide some indication as to the potential significance of protein damage. Kinetic data also provide indications (but only this) of the major targets of different biological oxidants, both radical and two-electron (Table 6). It should however be noted that the *extent of damage* and its *importance* are not necessarily equivalent–limited damage to a critical target, may have much greater effect than massive damage to redundant or unimportant sites.

**Table 6:** Overview of protein modifications induced by reactive oxidants

Oxidant	Major sites of reaction
HO^•^	All residues
RO^•^	Most residues
ROO^•^	Cys, Met, Trp, Tyr
CO_3_^−^•^^	Cys, Met, Tyr, Trp, His
NO_2_^•^	Cys, Tyr/Trp radicals
O_2_^−^•^^	Superoxide dismutase, some transition metal ions, Fe–S clusters, Tyr/Trp radicals
^1^O_2_	Cys, Met, Trp, Tyr and His
HOCl/HOBr	Cys, Met, cystine, His, α-amino group, Lys, Trp
Peroxynitrous acid (ONOOH)	Cys, Met, Tyr, Trp, selenocysteine
UVB light	Trp, Tyr, cystine
Reactive aldehydes	Cys, Arg, Lys, His, α-amino group
HOSCN	Cys, selenocysteine
H_2_O_2_	Cys, selenocysteine

In the following sections the chemistry and mechanisms of damage to proteins induced by some radical and non-radical (two-electron) oxidants are discussed. This cannot be all encompassing, but is intended to provide an overview and key references. This is followed by a more detailed discussion of protein peroxidation–the formation and role of (hydro)peroxides on amino acids and proteins–a major pathway in protein modification.

## RADICAL REACTIONS WITH PROTEINS

Radicals can undergo hydrogen abstraction, electron transfer (oxidation or reduction), addition, fragmentation and rearrangement, dimerization, disproportionation and substitution (concerted addition and elimination) reactions with amino acids, peptides and proteins. These reactions have been extensively reviewed (e.g. [7,9–27]).

As a there are 20 common amino acid side chains as well as the peptide backbone, a large number of different radicals can be generated on proteins. Damage to free amino acids, also occurs, but these species are usually present at lower concentrations (micromolar) than the side chains of proteins (high millimolar), so free amino acid damage may be *quantitatively* less abundant (though not necessarily of less importance).

The radicals formed depend critically on the nature and reactivity of the attacking radical. Electrophilic (electron-deficient) radicals are more common than nucleophilic radicals *in vivo*, and these radicals (which include HO^•^, and other oxygen-derived radicals such as alkoxyl RO^•^ and peroxyl ROO^•^), preferentially oxidize electron-rich sites. A major pathway is hydrogen atom abstraction from C–H (or S–H with Cys) bonds. For aromatic amino acids side-chain addition to the ring predominates, whereas with Met and cystine adduct formation at the sulfur occurs. Nucleophilic radicals (e.g. phenyl and some other carbon-centred species) preferentially attack electron-deficient sites. The positional selectivity of radical attack is well understood for free amino acids [7,9–27]. The second order rate constants, *k* for HO^•^ with free amino acids ranges between 10^7^ (Gly) and 10^10^ M^−1^·s^−1^ (Trp, His and Cys) [28], with preferential attack at sites remote from the electron-withdrawing (deactivating) protonated amine group. This deactivating effect is negated on incorporation of the amine into a peptide bond, resulting in a different distribution of species between amino acids and peptides. For *free* amino acids side-chain damage predominates over attack at the α-carbon. The effect of the amine group decreases with distance, so for amino acids with large side-chains (Val, Leu, Ile) damage is skewed towards remote side-chain sites, with this affected by both the number of available C–H bonds (i.e. statistical factors) [29–32], and the stability of the resulting carbon-centred radicals, with tertiary (i.e. RR′R′′C^•^) > secondary (RR′CH^•^) > primary (RCH_2_^•^) [32–36]. The selectivity of attack is further affected by functional groups that can stabilize/destabilize radicals. Thus hydrogen atom abstraction occurs preferentially adjacent to the–OH groups of Ser and Thr [37]. In contrast the protonated side-chain amine of Lys disfavours attacks at C-6, with abstraction occurring predominantly at C-4 and C-5 [33,34,38,39]. In each case, these reactions generate carbon-centred radicals whose fate is discussed below.

Addition reactions are typically faster than hydrogen atom abstraction reactions, due to the more favourable transition state energies, and hence addition to Phe, Tyr, Trp and His, and the sulfur atoms of Met and cystine, predominates over C–H bond hydrogen atom abstraction [40,41]. The resulting adducts undergo a range of subsequent reactions, with these including formation of peroxyl radicals and hydroxylated and carbonyl-containing products [7,24,37,42,43].

Hydrogen abstraction from the S–H (thiol) group of Cys is fast and gives thiyl radicals (RS^•^) [12,44–46]. The chemistry of these radicals is complex (reviewed [47–49]) but includes hydrogen atom abstraction, both intra- and inter-molecularly, from suitably positioned C–H bonds to give carbon-centred radicals. Thus initial oxidation at the Cys thiol can result in subsequent carbon radical generation at both α- (backbone) and β-carbon (side-chain) sites of the same amino acid (via formal 1,3- and 1,2-hydrogen shifts) as well as at neighbouring amino acids (both in terms of sequence and spatially) [48,49]. Subsequent reaction of these carbon-centred radicals with molecular O_2_ (to give ROO^•^) can “fix” damage at the carbon site.

Reaction of free Met with HO^•^ results (predominantly) in adduct formation at sulfur atom, with this species undergoing complex subsequent reactions involving the free amine and carboxy group that result in degradation of the amino acid [50–52]. Limited hydrogen-atom abstraction also occurs at the C–H bonds adjacent to the thioether centre, with the resulting carbon-centred radicals undergoing rapid reaction with O_2_ [53,54]. These hydrogen atom abstraction processes are of greater significance with electrophilic radicals other than HO^•^. In proteins, a major product of the initial adduct is the sulfoxide.

In proteins and peptides, hydrogen-atom abstraction also occurs at α-carbon C–H bonds. The resulting α-carbon radical is stabilized by electron delocalization on to the amide and the carbonyl functions [55,56] though the magnitude of this stabilization depends on the attached side chain due to steric and electronic interactions arising from the need for planarity for spin delocalization. This results in decreased stabilization of the α-carbon radical formed from amino acids with bulky side chains (e.g. Val) when compared with Gly despite the greater inherent stability of the tertiary α-carbon radical (from Val), over the secondary species formed from Gly [16,57–60]. The extent of backbone oxidation is also dependent on the local protein structure (helix, sheet etc.) with theoretical calculations indicating that α-carbon radical stability varies with secondary structure, as this constrains radical geometries, with a preference for α-carbon radical formation at Gly residues in antiparallel β-sheets [61–63]. Structural factors also limit access of attacking radicals to some sites, including the backbone. Side-chain reactions may therefore be of greater importance for globular or sheet proteins, than for disordered/random-coil peptides where more extensive backbone damage may occur.

Damage is more selective with less reactive radicals, as such reactions can have late transition states with significant radical character at the incipient radical site; as a result radical stabilizing factors become more important, resulting in a more limited number of species.

## SITE SELECTIVITY IN PROTEIN DAMAGE

Selective damage can arise from metal ion binding at particular side chains, with radical formation occurring in proximity to these residues [19]. Site-specific radical formation and formation of specific protein fragments has been observed with catalase [64,65], BSA [66] and β-amyloid precursor protein [67] among others. With thyrotropin-releasing hormone, copper complexation occurs at the His residue in a sequence ∼Glu-His-Pro∼, with HO^•^ abstracting a hydrogen atom from the α-carbon site of the Pro residue [68]. Site selective modification of His residues also occurs with glutamine synthetase [69,70] and human growth hormone [71]. Selective Met modification has been reported for peptides treated with Fe^3+^/O_2_/ascorbate [72], with both His and Met in human relaxin [73], and Trp in peptides exposed to Fe^3+^/O_2_^−•^ [74]. Comparison of data obtained for proteins exposed to radiolytic HO^•^ (i.e. no metal ions) with metal ion-generated radicals, has been reported to result in enhanced His, Cys, Met, Lys, Arg, Trp loss due to metal ion binding (reviewed [19]). Whether metal ion systems generate “free” HO^•^ in not always clear, with metal ion-peroxy, metal ion-oxo and high-oxidation-state metal ion complexes invoked in various cases [31,72,74–76]. Metal ion-hydroperoxide complexes (M*^n^*^+^-OOH) have been used to induce site-specific cleavage on proteins (e.g. [77–79]), with the metal ion localized by specific tethering, allowing the 3D structure in the vicinity of the metal ion to be probed [80,81]. Site-specific damage has also been proposed to occur as a result of the autoxidation (or metal-ion catalysed oxidation) of sugar molecules covalently-linked to proteins [82–86].

## FATE OF INITIAL AMINO ACID-, PEPTIDE- AND PROTEIN-RADICALS

The above data indicate that carbon-centred radicals are major initial intermediates in radical damage to amino acids and proteins. Similar radicals are generated via secondary reactions (e.g., rearrangement and fragmentation) of alkoxyl [87–89], peroxyl (reviewed [90]), thiyl [49,91] and nitrogen-centred radicals [92,93].

Dimerization and disproportionation are the major fates of carbon-centred radicals in the absence of O_2_ [94]. These reactions are structure-dependent, but typically very rapid due to their low energy barriers. Some dimerization products have been characterized (e.g. from α-carbon radicals generated from free amino acids and small peptides) [94,95]. With larger peptides, side-chain cross-links are observed, but the huge number of possible combinations, permutations and stereoisomers, makes analysis of these species highly challenging.

As such dimerization/disproportionation involves *two* radicals, the extent of these reactions is critically dependent on the radical flux, which is usually low in complex systems, and radical lifetimes. Long-lived radicals (e.g. phenoxyl radicals from Tyr and indolyl radicals from Trp) undergo dimerization with other radicals; 2*k* for Tyr phenoxyl radical self-dimerization is ∼5×10^8^ M^−1^·s^−1^ [96–98] with this giving both carbon–carbon and carbon–oxygen bonded species, with the former predominating. Quinone–nucleophile, Trp–Trp, and Trp–Cys species have also been reported [99–103]. Michael addition of nucleophiles to quinone products of aromatic amino acids appears to be particularly important (e.g. with DOPA-quinone from Tyr oxidation [104,105]). Cross-reaction of Tyr/Trp radicals with O_2_**^−•^**is also a major fate (see below).

Carbon-centred radicals can induce hydrogen-atom abstraction from weak X–H bonds (e.g. S–H bonds of thiols) and is of significance in low O_2_ environments (e.g. in tumours), when the radical is stabilized, and/or when dimerization is prevented by steric constraints (e.g. in proteins). This results in carbon-radical repair and formation of secondary species, such as thiyl radicals [45,106]. Repair of α-carbon (backbone) radicals by neighbouring Cys residues can result in L- to D-isomerization of amino acids [107] and dramatic effects on protein structure, function and immunogenicity [108,109]; the quantitative significance of such isomerization is unclear at present. Repair can occur both inter- and intra-molecularly (i.e. transfer from the Cys α-carbon site to a thiol, and the reverse, within peptides) [91,110–112]. Some carbon-centred radicals also undergo slow unimolecular elimination reactions. Thus α-hydroxyalkyl radicals with β-amino groups can release ammonia, a process that may be of significance with C5 radicals on 5-hydroxylysine, and for free Ser and Thr [113].

**Figure 3:**
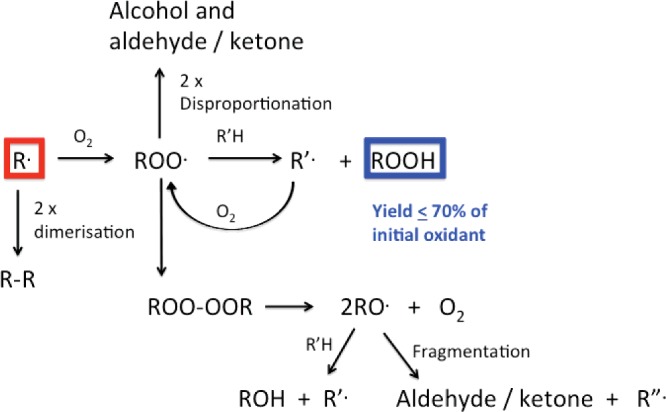
Overview of biological fates of carbon-centred (R^•^, highlighted in red) and peroxyl radicals (ROO^•^) in biological systems with subsequent formation of amino acid-, peptide- and protein-hydroperoxides (highlighted in blue)

Despite these other possible routes for carbon-centred radical removal, the predominant reaction in most situations is reaction with O_2_ to give peroxyl radicals (ROO^•^, Figure 3) as these reactions have *k* values near the diffusion-controlled limit (10^9^–10^10^ M^−1^·s^−1^) and the O_2_ concentration usually exceeds that of other radicals. O_2_ addition can be slow with highly delocalized radicals such as Tyr phenoxyl (*k* < 10^3^ M^−1^·s^−1^ [114]). With heteroatom-centred species (e.g. RS^•^) O_2_ addition can be moderately fast (*k* ∼ 10^8^ M^−1^·s^−1^), but is readily reversed [44]. Peroxyl radicals may also be generated from metal ion-catalysed decomposition of amino acid, peptide and protein hydroperoxides (see below [115,116]).

Peroxyl radicals can undergo multiple reactions (Figure 3). In biological systems with high concentrations of C–H and S–H bonds, or electron-rich species (from which the peroxyl radical can remove an electron and subsequently undergo protonation), *hydroperoxides* are major products. These species are discussed in depth below.

Peroxyl radicals can also undergo radical–radical termination reactions that directly yield alcohols and aldehydes/ketones; this can involve two ROO^•^, or ROO^•^ with HOO^•^/O_2_**^−•^**. For RCH_2_OO^•^ or RR′CHOO^•^, cross-reaction yields an alcohol and an aldehyde/ketone [117–121]. For tertiary peroxyl radicals (RR′R′′COO^•^) where dispropotionation cannot occur, tetroxides (RR′R′′COO-OOCRR′R′′) are formed that decay to give two RO^•^ and O_2_ [90,119,121]. Some of these reactions also yield ^1^O_2_ [90]. The resulting tertiary RO^•^ can carry out hydrogen-atom abstraction (or electron abstraction followed by rapid protonation) to give an alcohol, or undergo (particularly in aqueous solution) β-scission to give a ketone and another R^•^. These reactions are routes to protein carbonyls, a commonly used marker of protein oxidation [122,123]. However as these are *radical–radical* reactions, the carbonyl yield depends on the radical flux, so a fixed concentration of oxidant may give different yields depending on how rapidly the oxidant is generated.

Carbonyls are also generated from ROO^•^ that contain α-heteroatoms (α-hydroxyl or α-amino groups), as these undergo rapid unimolecular elimination of HOO^•^/O_2_**^−•^** [124–129]. Thus ROO^•^ at C-6 on Lys side chains eliminate NH_4_^+^ and HOO^•^ to give α-aminoadipate-δ-semialdehyde, a known marker of protein damage [130]. Similar reactions occur with ROO^•^ formed on the β-carbons on Ser and Thr, giving an aldehyde and ketone respectively. Analogous reactions occur during backbone fragmentation (see below). Unimolecular elimination of HOO^•^/O_2_**^−•^** is also of significance for ROO^•^ generated on aromatic rings after initial radical addition and subsequent O_2_ adduction [131]. In each case, fragmentation does not destroy the radical–products are formed *together with* a new radical that can undergo further reaction. These reactions may therefore contribute to chain reactions and damage propagation (cf. the chain reactions of lipid peroxidation).

## MECHANISMS GIVING RISE TO HYDROPEROXIDES (PROTEIN PEROXIDATION)

As most carbon-centred radicals react rapidly with O_2_, and there are an abundance of targets with which the resulting ROO^•^ can react, it is not surprising that hydroperoxides are major products. However as these species can undergo subsequent reactions, the true significance of these species is only now being appreciated.

The first report on protein peroxide formation appears to be from 1942 [132]. Later studies [133] showed that irradiated BSA could induce methacrylate polymerization, probably as a result of peroxides on the irradiated protein. Occasional reports on protein peroxides appeared in the radiation chemistry/biology literature up to the 1980s but in depth studies only began to appear in 1990s; early work is reviewed in [8].

**Figure 4:**
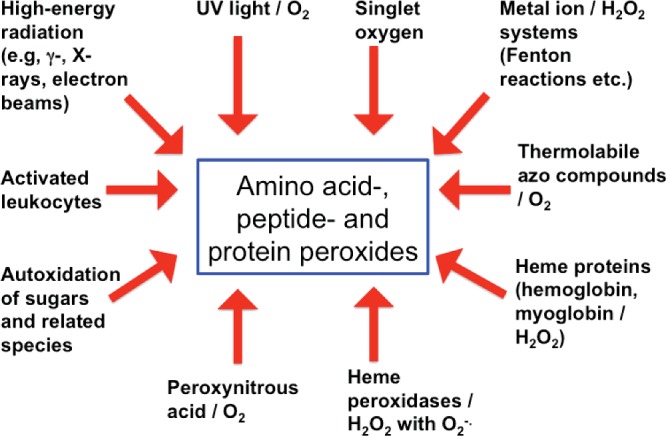
Summary of currently known oxidation systems that can give rise to amino acid-, peptide- and protein-hydroperoxides in the presence of molecular oxygen (O_2_) This list is unlikely to be exhaustive.

Steady-state irradiation methods have provided definitive evidence for amino acid-, peptide- and protein-hydroperoxides, as this methodology allows clean generation of defined amounts of radicals [8]. Manipulation of the reaction atmosphere has allowed the critical role of O_2_ to be proven. Early studies were confounded by the presence of both H_2_O_2_ and hydroperoxides in irradiated mixtures, but the demonstration of the high specificity of catalase for H_2_O_2_, and a lack of reaction with amino acid and protein hydroperoxides has resulted in easier quantification (see below) [8,134]. A large number of different oxidizing systems involving radicals and some two-electron oxidants (e.g. ^1^O_2_) are now known to yield amino acid-, peptide- and protein-hydroperoxides (Figure 4). The hydroperoxide yield with different oxidants is variable, due to other competing reactions, but there are limited examples where these are *not* detected, if a system is examined in detail using appropriate methods, and involves reactions where O_2_ is present.

## SELECTIVITY AND YIELDS OF AMINO ACID-, PEPTIDE- AND PROTEIN-HYDROPEROXIDE FORMATION

### Free amino acids

Free amino acids exposed to γ-irradiation (e.g. ^60^Co) in the presence of O_2_ can give high hydroperoxide yields with these being formed in a radiation dose-dependent manner [13,115,116,134–136]. Exclusion of O_2_ prevents their formation. The levels detected with all the common free amino acids are summarized in Table 7 [135], with the maximum levels corres-ponding to ∼40% of the initial HO^•^, under conditions where all the HO^•^ should be scavenged by the amino acids (>∼10 mM) [134,135]. These values may be underestimates, as hydroperoxide decomposition can occur during both continued irradiation (via electron attachment to the peroxide and cleavage to give HO^−^ and RO^•^), and via thermal degradation prior to and during analysis (as a consequence of the time needed to remove H_2_O_2_ using catalase). Some amino acids (Cys, cystine, Ser, Thr) give very low yields [135]. This is readily rationalized for Cys and cystine as reaction occurs preferentially at the sulfur centres. In addition, with each of these amino acids, ROO^•^ formed on the heteroatom-substituted carbon, undergo rapid elimination (see above) rather than the hydrogen atom/electron transfer to form hydroperoxides [12]. Aromatic amino acids give moderate yields, due to the occurrence of alternative reactions at the aromatic rings, though this is dependent on the radical and reaction conditions. High hydroperoxide yields are detected with HO^•^ and amino acids with large numbers of aliphatic C–H bonds (Val, Leu, Ile, Glu, Lys, Pro) from which hydrogen atom abstraction and subsequent ROO^•^ formation can occur [134,135]. Low yields of (backbone) hydroperoxides are formed on free Gly consistent with the relatively slow rate of hydrogen abstraction from the α-carbon site (see above). The hydroperoxides formed on Leu, Val and Lys have been characterized in detail [35,36,38], but in other cases these are less well defined.

**Table 7:** Efficiency of formation of hydroperoxides (number of hydroperoxide groups formed per HO^•^ × 100) on free amino acids (20 mM) exposed to 315 Gy of ^60^Co γ-radiation at pH 7.4 Data from [135].

Amino acid	Peroxidation efficiency
Valine	49
Leucine	44
Proline	44
Isoleucine	43
Lysine	34
Glutamic acid	28
Tryptophan	18
Glutamine	16
Arginine	13
Alanine	11
Aspartic acid	6
Phenylalanine	5
Histidine	4
Glycine	3
Tyrosine	3
Asparagine	2
Hydroxyproline	2
Cysteine	0.4
Methionine	0
Serine	0
Threonine	0

**Figure 5:**
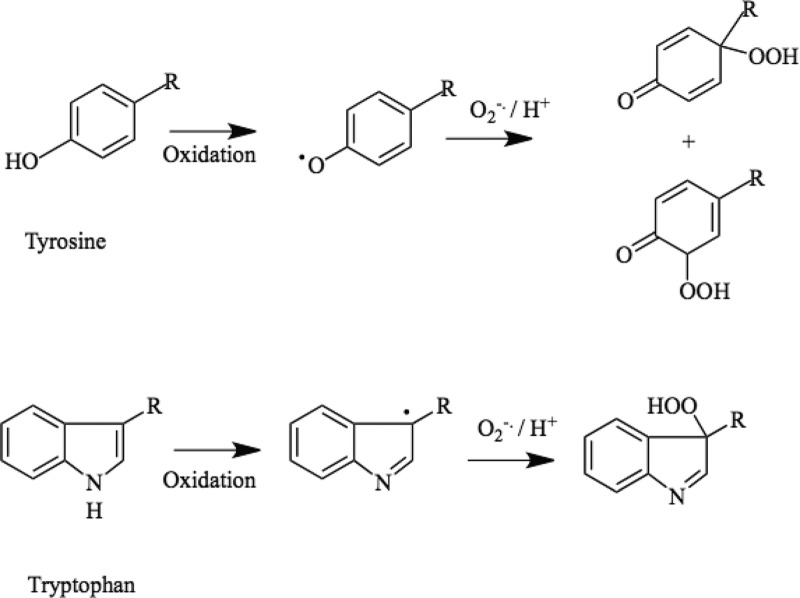
Formation of hydroperoxides on reaction of Tyr phenoxyl radicals and Trp indolyl radicals with the superoxide radical anion, O_2_^−^•^^ In the case of the Tyr-derived species, these hydroperoxides can undergo further reactions with nucleophiles, including thiol, amine and amide groups to give more complex structures as a result of the presence of the conjugated double bond and carbonyl group, which is a reactive Michael acceptor. The resulting structures may retain the hydroperoxide function (see text).

For Tyr, a major route to hydroperoxide formation is via reaction of an initial phenoxyl radical with O_2_**^−•^** (Figure 5). This dimerization has a low energy barrier resulting in rate constants near the diffusion limit, and is efficient due to the (relatively) long-lived nature of the parent radicals which increases their steady-state concentration and hence probability of reaction. These hydroperoxides are formed predominantly at C1 (the site of–CH_2_-attachment) and C3 (*ortho* to the–OH group), as these have the highest spin density, with the C1 species predominating as this tertiary hydroperoxide is of greater stability than the secondary hydroperoxide formed at C3 [98,137–143]. A C1 hydroperoxide has also been detected with HO^•^ from Fe^2+^/H_2_O_2_ [137]. These hydroperoxides can react with other nucleophiles, due to the presence of carbonyl-conjugated double bonds, which are good Michael acceptors, with data reported for adduction of thiol and amine groups to the ring to give both more complex hydroperoxides, and monoxides after hydroperoxide reduction (e.g. [138,140,141]). Similar processes (Figure 5) generate hydroperoxides from Trp indolyl radicals [144]. With both these amino acid radicals, O_2_**^−•^** addition predominates over electron transfer from O_2_**^−•^** to the oxidized ring (i.e. radical repair).

High yields of endo- and hydro-peroxides can also be formed by singlet oxygen (^1^Δg; ^1^O_2_) (reviewed [9,25,145]. ^1^O_2_ is formed by multiple chemical and physical processes including light-mediated reactions (Type 2 photochemical processes), enzymatic (peroxidase-, lipoxygenase- and cyclooxygenase- and haem-mediated reactions), cellular (e.g. from activated leucocytes) and chemical processes (e.g. reaction of H_2_O_2_ with HOCl, ozone-mediated reactions, to a limited extent in ONOO^−^/ONOOH reactions, termination reactions of peroxyl radicals). ^1^O_2_ reacts particularly rapidly by cycloaddition to aromatic rings, though addition to sulfur centres is also a significant reaction [146–148]; both processes can give peroxidic species.

**Figure 6:**
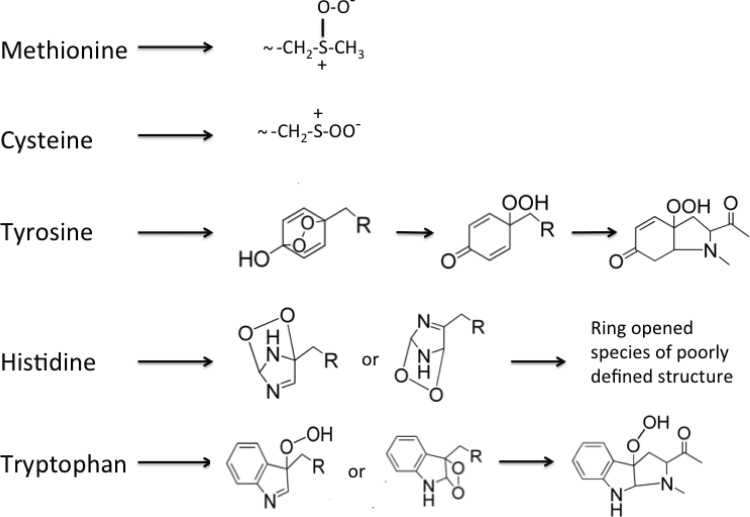
Peroxidic species identified on reaction of singlet oxygen (^1^O_2_) with reactive methionine, cysteine, tyrosine, histidine and tryptophan side chains Other species may also be formed, particularly with histidine (see text).

With Cys and Met, adduct formation by ^1^O_2_ is rapid and results in zwitterions with peroxide-like character (Figure 6) [149–151]. The subsequent reactions of these species are not fully understood, particularly in the case of proteins. With Cys, the RS^+^-OO^−^ species can give rise to disulfides (cystine, RSSR) as a major product, but formation of thiosulfinates and oxy acids also occurs [149,150]. The yield of these different species depends on the conditions and thiol concentration, factors that may have significant impact on the fate of these species when formed at isolated sites on proteins. Whether RS^+^-OO^−^ reacts significantly with other targets on proteins remains to be elucidated. For Met, the initial R_2_S^+^-OO^−^ adduct undergoes pH-dependent reactions that gives rise to two molecules of the sulfoxide with another molecule of parent, or a single molecule of sulfoxide and H_2_O_2_ via complex reactions [151].

For Tyr, Trp and His, ^1^O_2_ addition gives short-lived endoperoxides that can ring-open to give hydroperoxides at ring positions (Figure 6). These include hydroperoxides at C1 and C4 for Tyr, C3 for Trp and C2, C4 and C5 for His [144,152–160]. The structures of some of these species have been elucidated by MS and NMR (reviewed [145]). As ^1^O_2_ reactions with these side chains are fast and selective (when compared with HO^•^/O_2_ or some other radicals), hydroperoxide formation can be very efficient (i.e. high rates of conversion of ^1^O_2_ to hydroperoxide) and the absolute concentrations very high (e.g. millimolar).

### Peptides

With small peptides a similar pattern of side-chain hydroperoxide formation appears to occur (i.e. high hydroperoxide yields on side chains with large numbers of aliphatic C–H bonds), but increased yields of backbone hydroperoxides are detected, consistent with increased formation of initial α-carbon radicals. Thus although free Ala gives low hydroperoxide yields, moderate levels are detected with *N*-acetyl-Ala, and higher concentrations with (Ala)_3_, (Ala)_4_ etc. [116]. High levels have also been detected on peptides such as (Val)_3_ and larger species, and particularly those with high aliphatic amino acid contents [116,161,162].

### Proteins

Exposure of most proteins to oxidants generates peroxides at varying levels. Hydrogen or electron transfer and subsequent protonation reactions of ROO^•^, reaction of protein radicals with O_2_**^•−^**, and ^1^O_2_-mediated reactions are all major sources. The ROO^•^ pathway appears to predominate for aliphatic side-chain radicals, and the O_2_**^−•^** and ^1^O_2_ pathways for aromatic side chains. Zwitterionic peroxides are also likely to be formed from Cys and Met residues, but these have not been characterized on proteins.

Although species such as HO^•^ gives peroxides at multiple sites on a protein, a number of examples are known where hydroperoxide formation is highly specific–both with regard to amino acid type and location. Site-specific formation of Tyr phenoxyl radicals can result in hydroperoxides on specific residues via reaction with O_2_**^−•^** [140–142,163]. This offers exciting possibilities with regard to studying the chemistry of single well-defined peroxide species on proteins. Tyr hydroperoxide formation has been shown to compete effectively against other phenoxyl radical reactions, such as dimerization to give dityrosine, when the radical is isolated or electronically-hindered [140–142].

For Trp, evidence has been presented for addition of O_2_ to C3 radicals on the indole ring, as a result of the relatively high electron density at this site, resulting in the formation of C3 peroxyl radicals [164–167]. Subsequent hydrogen (or electron) transfer reactions to this ROO^•^ would result in Trp C3 hydroperoxides, in addition to the O_2_**^−•^** addition pathway. The O_2_ addition pathway may be limited to proteins, as O_2_ does not affect the rate of decay of free Trp indolyl radicals [144] indicating that *k* for O_2_ addition must be <∼10^5^ M^−1^·s^−1^, with the rate constant for dimerization of (free) Trp radicals being 7.3×10^8^ M^−1^·s^−1^ [144]. Whether related chemistry occurs with His-derived radicals to give hydroperoxides is unknown.

Although many of the hydroperoxides / endoperoxides formed on proteins are similar to those formed on amino acids, the decreased rate of radical–radical (termination) and radical–molecule (repair) reactions with proteins, due to steric and electronic effects, appears to increase hydroperoxide yields. Although radical–radical combination occurs with *k* ∼ 10^9^ M^−1^·s^−1^ for low-molecular-mass radicals, and hence can be a major removal pathway, these processes are often considerably slower for protein radicals [168], with this resulting in an increased extent of O_2_ addition to carbon-centred radicals to give ROO^•^ and hence hydroperoxides, and O_2_**^−•^** addition to Tyr phenoxyl and Trp indolyl radicals. Similar arguments apply for some of the species generated by ^1^O_2_ (e.g. Cys and Met). Disproportionation and dimerization of pairs of ROO^•^ are also likely to be more limited with proteins, and the rate of hydrogen (or electron) abstraction reactions to give hydroperoxides higher due to the high concentration of available C–H bonds within proteins.

Whereas the above factors may enhance hydroperoxide yields and lifetimes, other factors that may have the reverse effect, including enhanced reaction with, for example, neighbouring Cys and Met residues that may reduce hydroperoxides to alcohols, or form adduct species (e.g. with GSH [141]). Such reactions are well established *intermolecular* peroxide removal pathways (see below), but there is also evidence for *intramolecular* processes within proteins [142].

## DETECTION AND QUANTIFICATION OF AMINO ACID-, PEPTIDE- AND PROTEIN-HYDROPEROXIDES

Hydroperoxides can be quantified by classical titration (e.g. using KMnO_4_, iodometric or Ti^3+^), and although this approach can be used with isolated amino acids and peptides, these methods are less appropriate for complex systems due to competing reactions. Iodometric titration is reported to be the most accurate [169], but this is technically complex due to the requirement for anaerobic conditions, which can be difficult to achieve for cellular and complex systems.

A widely used and technically simple method is the FOX (ferric iron–xylenol orange) assay, where the hydroperoxide oxidizes Fe^2+^ to Fe^3+^ which forms a complex with Xylenol Orange that absorbs strongly at 560 nm [170,171]. In the original assay [170], sorbitol was added to enhance the absorbance readings via chain reactions, but as the chain length appears to be variable, this form of the assay is no longer widely employed [172]. A number of protocols are available [171,173–175]. This approach has been developed to allow hydroperoxide separation by HPLC prior to on-line detection–this method can give data on approximate concentrations of individual species with a sensitivity limit of 10–25 pmol [162]. One limitation of this assay is the unknown stoichiometry of the Fe^2+^-peroxide reaction. Although this should be 2:1, higher values have been reported, and this is peroxide- and protein-dependent [171]. As these values are not known, and not easy to determine, peroxide yields are usually reported as H_2_O_2_ equivalents [171]. Although this is not a major problem in comparative studies, accurate mass balance becomes impossible. Furthermore, like other approaches, an implicit assumption is made about access to all the hydroperoxides present, and this may not always be correct, particularly with proteins; buried hydroperoxides may react slowly (or not at all) resulting in an underestimation of peroxide levels.

Chemiluminescence using microperoxidase and luminol has been used to quantify peroxides [136]. Although this method is sensitive (50 pmol detection limit), this technology suffers from some of the same problems as the FOX assay with regard to limited understanding of the stoichiometry of the light-generating reactions. Consequently the values obtained may not be absolute concentrations, and buried peroxides may not react rapidly with microperoxidase, which is considerably more sterically-bulky than the Fe^3+^ used in the FOX assay.

Hydroperoxides can be detected by MS approaches as they yield distinctive *m/z* +32 peaks (e.g. [153–157,161,162]). However peroxide instability under MS conditions (particularly elevated temperatures) makes absolute quantification complex. MS is however very valuable in determining the *sites*, and *identities* of peroxides on complex molecules, though considerable development and refinement of the technique will be required to determine absolute concentrations at particular locations. MS approaches can also be used to determine the sites (and yields) of alcohols (*m/z* +16) from hydroperoxide decomposition [35,36,38,137,161]. This possibility, when coupled with immediate sample reduction to convert any hydroperoxides to the corresponding alcohols, has allowed information to be obtained as to hydroperoxide yields coupled with location information [161]. Although this is potentially a valuable method, it is limited by the assumption that hydroperoxides are the primary precursors of the detected alcohols. This is unlikely to be completely correct, as dismutation reactions of ROO^•^ and hydrogen abstraction by RO^•^ can also generate these species. However as RO^•^ can arise from hydroperoxides (via one-electron) reduction the data may not be perturbed as much as might be initially thought.

Reaction of hydroperoxides with (non-fluorescent) coumarin boronic acid probes, to release fluorescent products (e.g. 7-hydroxycoumarin), may be an alternative and useful means of (semi-) quantifying hydroperoxides when coupled with a standard curve constructed using the authentic fluorophore [176]. This approach can be used for high throughput studies, and also allows the kinetics of oxidation to be examined [176]. However, as with many of the other approaches described above, there is a lack of authentic hydroperoxide standards, and uncertainty as to whether all the peroxides present undergo reaction.

## STABILITY OF AMINO ACID-, PEPTIDE- AND PROTEIN-HYDROPEROXIDES

**Figure 7:**
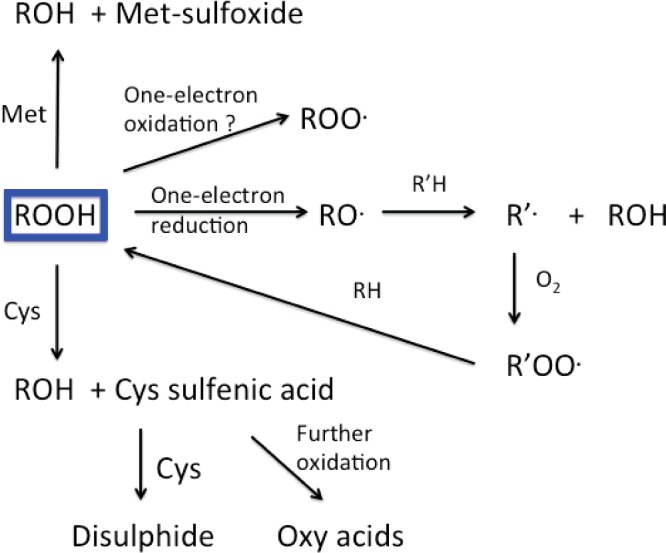
Overview of one- (radical) and two-electron (molecular) reactions of amino acid-, peptide- and protein-hydroperoxides (highlighted in blue) The two-electron reactions occur predominantly with Cys residues to give sulfenic acids, disulfides, and higher oxy acids. Reaction has also been reported for Met, some disulfides such as lipoic acid (not shown) and selenium-containing compounds, including selenomethionine and selenocysteine (Sec)-containing enzymes such as thioredoxin reductase and glutathione peroxidase (not shown). One-electron reduction yields alkoxyl radicals and further oxidation reactions (for further details of specific mechanisms see Figure 8), whereas one-electron oxidation may yield peroxyl radicals; the latter process is poorly characterized.

Amino acid-, peptide- and protein-hydroperoxides can have lifetimes of hours–weeks when kept at room temperature in the dark, and in the absence of metal ions, reductants, and other reactive species (e.g. enzymes) [134]. For some proteins, ∼30% of the initial hydroperoxide remained after 1 week at room temperature [134], but a half-life of ∼1.5 days has been estimated for some other protein hydroperoxides [135]. Lower temperatures enhance lifetimes, and these can be months–years at −20 or −80°C. Elevated temperatures, redox active metal ions (e.g. Cu^+^, Fe^2+^ [115,116,134,177,178]), UV light [136], or reductants (e.g. dithionite, triphenylphosphine, ascorbate, GSH, sodium borohydride [134,135]) can induce rapid decay (Figure 7). NADH and NADPH are ineffective in the absence of associated enzymes [134]. The most stable peroxides appear to those present at tertiary carbon sites, or sterically-isolated on proteins [8,115,116,135]. Some protein-derived hydroperoxides can survive enzymatic digestion by proteolytic enzymes such as pronase [179], thereby allowing initial high-molecular-mass species to be converted to smaller fragments or free amino acids for analysis; such reactions have potentially important implications for the cellular processing of ingested damaged proteins. The percentage recovery from such treatment is unknown (and is likely to be species dependent), due to problems with absolute and accurate quantification of hydroperoxides (see above).

## EVIDENCE FOR AMINO ACID-, PEPTIDE- AND PROTEIN-HYDROPEROXIDE FORMATION *IN VITRO* AND *IN VIVO*

There is increasing evidence, both direct and indirect, for the formation of hydroperoxides in complex systems. The exact identity of these is however less clear, and quantification can be problematic due to the ready decomposition or reaction of these species. Exposure of isolated low-density lipoproteins to O_2_**^−•^** generating systems [180] or human macrophage-like THP-1 cells has been reported to yield hydroperoxides on the apolipoprotein B100 protein, as well as lipid hydroperoxides [181]. The formation of these species was inhibited by the radical-scavenging antioxidant 7,8-dihydroneopterin [181].

Exposure of intact cells to oxidants generates protein hydroperoxides, though the yield (and presumably proteins involved) varies with the cell type. Exposure of mouse myeloma (Sp2/0-Ag14), U937 (monocyte-derived) and HL-60 cells to HO^•^, generated by γ-irradiation, generates protein hydroperoxides [182–184]. The concentration of these species within the cells has been reported to vary between 1 and 2 μM (in 2×10^6^ Sp2/0 cells [183]) to up to 1–2 mM in HL-60 cells [184]. These protein hydroperoxides were detected with irradiation conditions under which no lipid or DNA damage could be determined (though this is likely to be due to a limitation of the methods employed). These data have been interpreted as indicating that proteins are the major initial targets for radiation-generated radicals, and that protein peroxidation precedes significant lipid and DNA damage [182,183]. The yield of the protein-derived species was independent of the culture medium used suggesting that only radicals generated in close proximity to, or within cells are the peroxide-generating species [183]; this is in accord with the limited diffusion of HO^•^. In studies using HL-60 cells [184] the formation of the protein hydroperoxides showed a lag phase, ascribed to the action of endogenous antioxidants and particularly reduction by GSH which was lost concurrently; this interpretation is supported by supplementation studies with *N*-acetylcysteine (which decreased hydroperoxide levels), and L-buthionine sulfoximine (which depletes GSH, and enhanced hydroperoxide concentrations) [184]. Increased intracellular ascorbate levels were also reported to decrease hydroperoxide formation [184].

Other studies with HL-60 cells indicate that ROO^•^ (from the thermally labile azo compound AAPH, and O_2_) also generate protein peroxides in preference to lipid peroxides, with these present at concentrations of up to 3 μM per 3×10^7^ U937 cells [185]. The protein hydroperoxides had a half-life of ∼4 h at 37°C, and were formed without a lag phase [185], suggesting that cellular antioxidants cannot prevent the formation of these species (or more likely, a sub-population of them), and that the removal of these species once formed is inefficient (see below). Studies with human monocyte-derived macrophages have however, provided opposing data. In this case, AAPH/O_2_ exposure gave both lipid and protein hydroperoxides, with the former predominating [186]. This has been ascribed to the higher lipid content of these cells, and possible localization of peroxide formation to particular sub-cellular locations.

Photolytic reactions can generate protein peroxides in both cell lysates and intact cells [162,187,188]. Illumination of murine macrophage-like (J774A.1) or human monocyte (THP-1) cell lysates with visible light in the presence of the photosensitizer Rose Bengal and O_2_, generates multiple protein peroxides; the concentration of these peroxides has been reported to be up to ∼1.5 nmol per 10^6^ cells, or 10 nmol/mg cell protein [162,187,188]. In one study, the hydroperoxides were separated by HPLC before detection (using an on-line FOX reaction) and shown to be present on multiple proteins, though these were not identified [162]. These studies, together with those with THP-1 cells, indicate that protein hydroperoxides can be formed under conditions where the cells remained viable, as determined using ethidium bromide-binding to released DNA (a late marker of damage) [188] or lactate dehydrogenase release (a marker of membrane damage) [187]. The peroxides were predominantly present on trichloroacetic acid-precipitatable material (i.e. protein-derived), and not diminished by hexane–methanol extraction (i.e. not soluble in organic solvents, as lipid peroxides are). The peroxide yields were enhanced in D_2_O, and decreased by sodium azide, consistent with a mechanism involving ^1^O_2_ [187,188]. The peroxide levels detected in these experiments were not affected by pre-loading the cells with ascorbate, suggesting that this antioxidant does not markedly affect protein hydroperoxide concentrations generated by ^1^O_2_ under these conditions, in contrast with the γ-irradiation/HO^•^ studies discussed above [184]. With cell lysates prepared from THP-1 cells, identical peroxide yields were detected at short illumination times as seen with the intact cells, but higher yields were detected at longer times, consistent with (unknown) limiting factors in the intact cells [188].

Evidence has also been provided for peroxide formation on endogenous or exogenous proteins by cell-mediated reactions. Stimulated neutrophils can generate hydroperoxides on added free Tyr [189] or enkephalins [163], via myeloperoxidase-mediated reactions, involving phenoxyl radical generation from Tyr and subsequent cross-reaction with O_2_**^−•^**, to give a Tyr-derived hydroperoxide [163]; peroxide formation predominated over phenoxyl radical dimerization to give di-Tyr.

Hydroperoxide formation has been reported in plasma, with peroxides detected on fresh human plasma proteins illuminated with visible light in the presence of O_2_ and the photosensitizers Rose Bengal [162] or haematoporphyrin (J.A. Silvester, unpublished work). In the former study the concentrations of hydroperoxides detected were up to 75 μM in plasma that had been diluted to a protein concentration of 10 mg ml^−1^ [162]. HPLC fractionation of the photo-oxidized plasma indicated that the majority of the peroxides co-eluted with human serum albumin, consistent with the high abundance of this protein in plasma, and/or binding of the sensitizer to this species and localized peroxide formation. The latter explanation is supported by studies with other sensitizers that bind to serum albumins [190,191].

Little direct evidence is available yet for hydroperoxides in intact tissues, either normal or diseased. This may reflect the short half-lives and/or reactivity of these species. However there is considerable *indirect* evidence for their formation, particularly from the presence of degradation products. The detection of high levels of carbonyls and alcohols in both normal and diseased tissue specimens [122,192] is consistent with the formation of hydroperoxides, with the yield of these species typically being in the low nmol/mg protein range in most cases, though some higher values have also been reported (see e.g. [193]). Although there is good evidence that hydroperoxide degradation yields these products, the quantitative significance of each pathway is unknown, though it has been shown in some *in vitro* systems that this can be near quantitative [194]. As protein ROO^•^ dimerization is likely to be slow, it is likely that some protein ROO^•^ formed *in vivo* will yield hydroperoxides, with these subsequently decomposing to alcohols and carbonyls. This is clearly an area that warrants further investigation, but it is worth noting that strong evidence has been reported for alcohols being major products formed from hydroperoxides by biological reductants [35,36,38,194]. The evidence and detection of protein carbonyls in healthy and diseased tissue samples has been the subject of multiple reviews and will not be covered further here (e.g. [1,192,195–201]). Elevated levels of alcohols have also been detected in a range of tissue samples including atherosclerotic lesions [202], cataractous and normal aged lenses [203], and samples from people with diabetes [82].

The absolute levels of Val and Leu alcohols have been reported to be between 50 and 100 μmol per mol parent amino acid in human atherosclerotic lesions (corresponding to 1–4 pmol/mg wet mass of intimal tissue) [202], and 200–400 μmol oxidized amino acid per mole parent amino acid in advanced human lens cataracts (60–120 pmol oxidized amino acid/mg of dried lens tissue) [203]. These data allow a rough calculation to be made of the total flux of hydroperoxides to which such tissue proteins have been exposed. If it is assumed that the average protein concentration in cells is 5 mM (see earlier), that each protein contains on average 200 amino acids (i.e. a total amino acid side-chain concentration in cells of 1 M) and that Val and Leu account for between 10 and 20% of the side chains (cf. data in Protein Data Bank database), then the total exposure (assuming 100% reduction of the hydroperoxides to alcohols) would be 50–100 μmol hydroperoxides for the proteins in advanced atherosclerotic lesions, and 200–400 μmol hydroperoxides for the lens cataract proteins. The overall concentration is likely to be higher than this, as this calculation only considers products from Val and Leu of the multiple amino acids (Table 7) on which hydroperoxides can be generated.

## SECONDARY REACTIONS OF HYDROPEROXIDES: ONE-ELECTRON PROCESSES

**Figure 8:**
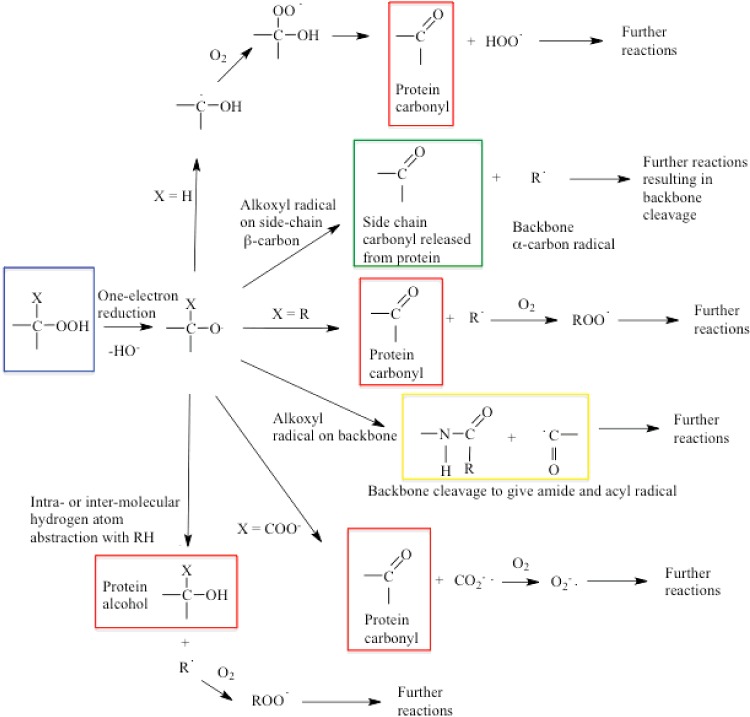
Secondary fragmentation, rearrangement and hydrogen atom abstraction reactions of alkoxyl radicals (RO^•^) generated from amino acid-, peptide- and protein-hydroperoxides Decomposition of hydroperoxides (highlighted in blue) to RO^•^ can result in stable protein products (carbonyls and alcohols, highlighted in red), loss of protein side chains as low-molecular-mass carbonyls (highlighted in green), backbone fragmentation (highlighted in yellow), as well as further reactive radicals than can propagate damage and chain reactions.

Alkoxyl radicals (RO^•^) can be formed on one-electron reduction of hydroperoxides (e.g. by transition metal ions via pseudo Fenton reactions), or by photolytic (e.g. short wavelength UV light) or thermal homolysis of hydroperoxides. RO^•^ undergo rapid addition and hydrogen abstraction reactions, and facile unimolecular fragmentation and rearrangement reactions which may enhance or propagate damage (Figure 8) [12,87,88,204,205].

Primary and secondary RO^•^ from hydroperoxides undergo rapid (formally 1,2-, but involving solvent molecules) hydrogen shift reactions in aqueous solution (*k* 10^6^–10^7^ s^−1^) to give α-hydroxyalkyl radicals [87–89,206]. These reactions compete with hydrogen abstraction to give alcohols [88,206]. With tertiary RO^•^, where 1,2-shift reactions are impossible, rapid β-fragmentation (*k* ∼ 10^6^ s^−1^) yields carbon-centred radicals and carbonyls (aldehydes/ketones) [204,207–209]. These reactions may result in damage transfer between sites in an amino acid or protein. It has been shown that a side-chain (β-carbon) RO^•^, formed from degradation of the corresponding hydroperoxide, can fragment to give both a backbone α-carbon radical (as a result of the stability of the latter) and a low-molecular-mass carbonyl from the side chain (Figure 8) [210,211]. These reactions result in modification of the initial side chain (which is lost from the protein) *and* the release of a further radical. These are therefore damage propagation (chain) reactions (see also below). β-Scission of RO^•^ formed from a hydroperoxide at C-4 on Glu side chains results in the loss of the adjacent side-chain carboxy group as CO_2_**^−•^**, and formation of an aldehyde (Figure 8) [115]. CO_2_**^−•^** is a powerfully reducing radical and reacts rapidly with O_2_ to give O_2_**^−•^**, thereby potentially propagating radical chains. Other hydrogen atom shift reactions can potentially transfer damage to the α-carbon site or other locations in proteins (Figure 8). RO^•^ formed at C-5 on Lys, Arg, Ile and Leu may abstract an α-carbon hydrogen atom via an intra-molecular 1,5-hydrogen atom shift though direct evidence for this process in proteins is lacking; such reactions are however facile in model compounds (*k* ∼ 8×10^6^ s^–1^) [89,212]. 1,6-shift reactions are less favourable and 1,4- and 1,3-shifts are rare unless solvent assisted; such reactions are however known for thiyl radicals [112,213–215].

## ROLE OF HYDROPEROXIDES IN PEPTIDE BACKBONE CLEAVAGE

With Gly-containing peptides, hydrogen atom abstraction by HO^•^ from the α-position (i.e. the backbone -CH_2_-) is a major process [31]. With small Ala-containing homopeptides α-carbon hydrogen abstraction can account for >90% of initial HO^•^ attack due to the greater stability of the α-carbon radical compared with the (primary) alkyl radicals generated from the methyl side chain [31]. With larger peptides the yield of backbone-derived carbon-centred radicals is lower, particularly when reactive side chains are present [13,31,161]. Subsequent reaction of these α-carbon radicals with O_2_ gives backbone ROO^•^ and hydroperoxides. Both species undergo further reactions that result in backbone cleavage (reviewed [7,13,37]). Backbone ROO^•^ can eliminate HOO^•^ to give imines that then undergo hydrolysis to the corresponding amides and carbonyl compounds. Studies on cyclo(Gly_2_) and cyclo(Ala_2_) have shown that these reactions are slow at neutral pH values, but more rapid at higher pHs where base-catalysed elimination of O_2_^−•^ occurs [216].

Decomposition of α-carbon (backbone) hydroperoxides (e.g. catalysed by metal ions or UV light) yields RO^•^ [115,116] which undergo β-scission to give a carbonyl group and a secondary acyl radical [^•^C(O)NHR] when the hydroperoxide is present within a peptide chain (i.e. remote from either backbone termini) [116]. This results in cleavage of the backbone. Carboxyl-terminal hydroperoxides give rise to alkoxyl radicals that release CO_2_**^−•^** [or ^•^C(O)NH_2_ in the case of a C-terminal amide]. This hydroperoxide/RO^•^ pathway appears to compete with the imine hydrolysis route to backbone fragmentation [7,13,37]. Backbone hydroperoxides therefore appear to be significant intermediates in peptide fragmentation, but the *quantitative* contributions of these two pathways is unknown. It is however established that fragments with new N-termini can be generated from oxidized proteins, with such termini consistent with the RO^•^-mediated fragmentation pathway [77,217–219]. Fragments consistent with this pathway are also generated during backbone cleavage of the R1 sub-unit of ribonucleotide reductase involving an α-carbon Gly radical [220].

## ROLE OF HYDROPEROXIDES IN PROTEIN CHAIN REACTIONS (PROTEIN PEROXIDATION)

Quantification of initial radical yields and amino acid loss in irradiation studies has provided evidence for a greater loss of amino acids than initial radicals formed [221]. This is consistent with *chain reactions* with each initial radical giving rise to secondary species that consume additional amino acids. Chain lengths of up to 15 have been reported if calculated on the basis of the initial yields of HO^•^ for isolated irradiated proteins [221] and up to 10 hydroperoxide groups per initial radical in HL-60 cells [184]. Although these chain lengths are modest when compared with lipid peroxidation (where values of >100 occur), it would appear that protein chain oxidation reactions can occur under certain circumstances and with both isolated proteins, and in intact cells. These are likely to involve some of the fragmentation and hydrogen atom abstraction reactions of ROO^•^, RO^•^ and hydroperoxides discussed above, but the chain-carrying species remain to be defined. Although hydrogen abstraction can also occur with R^•^, this may not result in additional amino acid loss as these reactions only *transfer* the damage and can regenerate the parent amino acid. However, if the transfer reaction results in a change in *stereochemistry* of the original amino acid this may result in significant changes in conformation and result in dysfunctional materials. This possibility is of major potential importance with backbone α-carbon radicals where inversion of the (usual) L-stereochemistry to D- can occur as a result of H-atom transfer to either face of the (planar or near planar) intermediate radical. Such inversion may have dramatic effects on secondary and tertiary structure. Similar processes can occur with the limited number of side-chain stereochemical centres. Stereochemical inversion has been shown to occur in a limited number of studies [107,108,111], but may be a common process, as sensitive methods to allow the examination of these reactions have only recently been developed. The low extent of O_2_ consumption detected during the studies of chain processes on isolated proteins (∼2 mol per mole attacking radical [221]) would only account for modification of four amino acid residues to alcohols or carbonyls, per initial radical, suggesting that fragmentation reactions must play a key role, as these processes can result in amino acid alteration without (necessarily) involving oxygen incorporation.

## SECONDARY REACTIONS OF HYDROPEROXIDES: TWO-ELECTRON REDUCTION BY LOW-MOLECULAR MASS SPECIES

**Figure 9:**
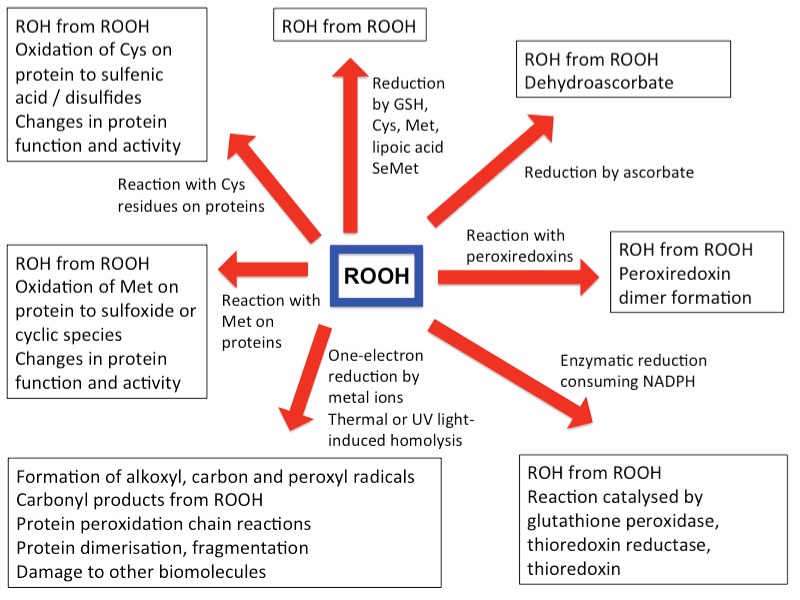
Potential fates of hydroperoxides present on amino acids, peptides and proteins in biological systems For further details see text.

Although it has been established that hydroperoxides can decompose to give further radicals, the importance of this pathway relative to two-electron reactions is unclear. Considerable evidence has been presented for rapid reaction of amino acid-, peptide- and protein-hydroperoxides with thiols (RSH) and thiolate anions (RS^−^), with the latter being more rapid. Reaction with free Cys and GSH is well documented [134,135,179], with the latter reaction being proposed as a major detoxification route in cells and *in vivo* [194] (Figure 9). Reaction with other thiols and related species has also been reported [179]. Disulfides react less rapidly, with little consumption of peroxides detected on extended incubation, with the exception of lipoic acid which appears to be much more reactive due to the presence of the strained five-membered ring [179]. Intermolecular reaction with free Met is inefficient, but limited reduction has been detected with the related species 3,3′-thiodipropionic acid [179]. In contrast, significant Met oxidation has been detected when the hydroperoxide and Met are in the same peptide, or in close physical proximity [163]. Thus Tyr hydroperoxides present on enkephalins readily oxidize Met residues to the sulfoxide, with the Tyr residue converted to a bicyclic species via adduction of the N-terminal amine to the aromatic ring [163]. This may be due to differences in hydroperoxide reactivity, or an increased rate of reaction due to the intra-molecular nature of the latter reaction that would be more favourable entropically.

Ascorbate is an effective hydroperoxide reductant [134,135,184], and dehydroascorbic acid has also been reported to react, but ascorbate-2-phosphate was ineffective [179]. Seleno compounds (ebselen and selenomethionine) are also highly effective hydroperoxide removal agents [179,222], though contradictory data for ebselen has been reported [223]; the reason for this discrepancy is unclear. With selenomethionine, which is oxidized to the selenoxide, removal of hydroperoxides appears to be *catalytic* when GSH and various enzymes are also present due to efficient subsequent reduction of the selenoxide ([222] and below).

With peptide hydroperoxides evidence has been obtained for concurrent loss of both hydroperoxides and thiols in cell lysates [179], consistent with data suggesting that GSH is a major removal agent for (at least some of) these species in cells [184,194]. Much less rapid, and less efficient, hydroperoxide removal and concomitant thiol oxidation, was detected with BSA hydroperoxides, possibly for steric reasons [179]. This is supported by data for BSA hydroperoxides pre-treated with Pronase to degrade the protein to smaller peptides; this resulted in both more rapid peroxide removal and increased thiol loss [179]. These data suggest that the long-lived hydroperoxides detected in intact cells (see above) may be present on large proteins, and/or are inaccessible to low-molecular-mass reductants such as GSH and ascorbate as a result of being (at least partly) buried within the protein structure.

Phenolic antioxidants (e.g. Trolox C, probucol, butylated hydroxytoluene) do not show any significant capacity to directly remove hydroperoxides, though they may scavenge radicals derived from them [179].

## ENZYMATIC REPAIR/REMOVAL OF AMINO ACID-, PEPTIDE- AND PROTEIN-HYDROPEROXIDES

The above data indicate that low-molecular-mass reductants can potentially remove some amino acid and peptide hydroperoxides in biological systems, with the slowest and least efficient reactions being with protein hydroperoxides. A similar situation appears to occur with enzyme-mediated removal but with more marked differences. There is little evidence for reaction of amino acid-, peptide- or protein-hydroperoxides with catalase, with this providing a useful means of distinguishing between these species and H_2_O_2_ [134,135,179]. No reaction occurs with superoxide dismutases, or peroxidases such as horseradish peroxidase, lactoperoxidase and myeloperoxidase, probably as a result of steric hindrance [179,223]. With ferric (met)myoglobin and haemoglobin reaction is slow [179], but more rapid reaction occurs with the oxy form [179]. Significant reaction occurs with oxyhaemoglobin from erythrocytes [224], with the reaction involving (overall) conversion of the oxy form to the met. These reactions are moderately fast with amino acid hydroperoxides, slower with peptide hydroperoxides, and large protein hydroperoxides (BSA- and lysozyme-derived) are essentially inert. The oxy to met conversion of haemoglobin induced by amino acid hydroperoxides has been reported to be faster than with H_2_O_2_; this was initially ascribed to possible trace levels of catalase in the haemoglobin preparations [224]. Later studies have shown that this is not a significant factor, and that this is a general phenomenon: many amino acid and peptide hydroperoxides are both *more reactive* than H_2_O_2_, and *poorly removed* by protective systems [179]. These data suggest that amino acid and peptide hydroperoxides may accumulate to much higher levels than H_2_O_2_ in cells and possibly be more damaging.

With the selenocysteine-dependent enzyme glutathione peroxidase (GPx), a similar pattern of reactivity is detected. With BSA and lysozyme hydroperoxides, the rate of peroxide loss is not enhanced in the presence of GPx plus GSH, over that detected with GSH alone [179]. In contrast, amino acid hydroperoxides and some peptide and small protein hydroperoxides (e.g. insulin hydroperoxide [223]) can be removed *catalytically* by a mixture of GPx and GSH, with more peroxide lost than with GSH alone [179,194,223]. Studies with phospholipid GPx (GPx4) have shown that this does not stimulate hydroperoxide decay, compared with GSH alone, with any of the hydroperoxides tested [223].

With selenomethionine (SeMet) both direct, and enzymatically enhanced hydroperoxide removal has been reported [225]. Direct reaction yields the selenoxide that can be readily converted back to parent SeMet by GSH as well as multiple reductase enzyme systems including NADPH/thioredoxin reductase and NADPH/thioredoxin/thioredoxin reductase [225]. Hydroperoxide removal occurred more rapidly with the enzyme systems compared with SeMet alone, and was near stoichiometric with respect to NADPH consumption and hydroperoxide removal, indicative of efficient enzymatic reduction [225]. Supplementation of cells with SeMet has also provided evidence for increased hydroperoxide removal relative to the unsupplemented cells, consistent with catalytic enzymatic removal of amino acid and peptide hydroperoxides, but not large protein hydroperoxides [225].

Peroxiredoxins 2 and 3, which are abundant Cys-containing cytosolic and mitochondrial enzymes that are maintained in their reduced state by the NADPH/thioredoxin/thioredoxin reductase system, rapidly reduce hydroperoxides. A number of hydroperoxides (formed by irradiation or photo-oxidation) have been shown to react rapidly with Prx2, with *k* between 10^2^ M^−1^·s^−1^ (for protein hydroperoxides) and 4×10^4^ M^−1^·s^−1^ (for low-molecular-mass species), with concomitant formation of disulfide-linked Prx2 dimers, and alcohols from the hydroperoxides [226]. Addition of leucine- and BSA-hydroperoxides to erythrocyte lysates resulted in Prx2 oxidation without significant GSH loss, indicating that Prxs are major intracellular targets for these hydroperoxides and have the potential to detoxify these species in cells. These reactions are not *repair* reactions, as they do not convert the oxidized amino acids back to the parent form (the reduction product being the alcohol), though they will limit secondary damage.

## BIOLOGICAL CONSEQUENCES OF AMINO ACID-, PEPTIDE- AND PROTEIN-HYDROPEROXIDE FORMATION

### Enzyme inhibition

As hydroperoxides react rapidly with many Cys residues, a number of studies have examined inhibition of enzymes that contain Cys residues, and particularly those with low p*K*_a_ values. The latter might be expected to react more rapidly due to the presence of a thiolate anion, RS^−^, which is a better nucleophile. Glyceraldehyde-3-phosphate dehydrogenase (GAPDH) is rapidly inactivated by a range of amino acid and protein hydroperoxides with the loss of activity associated with consumption of the protein thiols [227]. Little loss of activity was detected with decomposed hydroperoxides. The loss of activity was more rapid in some cases than observed with equal or higher concentrations of H_2_O_2_ [227]. This inactivation was not enhanced by the presence of Fe^2+^-EDTA (which catalyses radical formation from the hydroperoxides), consistent with *molecular* oxidation of a key Cys residue, but enhanced inactivation was detected with H_2_O_2_ and Fe^2+^-EDTA, consistent with contributions from both molecular and radical-mediated reactions in the latter case [227]. Glutathione reductase was less readily inactivated than GAPDH, but a loss of activity was still detected with high concentrations [227,228], whereas lactate dehydrogenase was unaffected [227]. Oxidation of key Cys residues by amino acid and protein hydroperoxides, coupled with a loss of activity, has been reported for papain and some cathepsin enzyme isoforms (B and L), but not others (D and G), with the former, but not the latter, being Cys-dependent enzymes [229]. These data are again consistent with Cys oxidation, and a sulfenic acid (RS-OH) intermediate has been detected with papain. Cellular caspases are also sensitive to inactivation by Tyr- and Trp-derived peroxides, but not with ovalbumin peroxides, with IC_50_ values for inhibition being ∼10 μM compared with 300 μM for H_2_O_2_ [230].

Inhibition of enzyme activity and associated Cys oxidation, has been detected with both isolated protein tyrosine phosphatases (PTPs) and these enzymes in cell lysates. The PTPs, together with protein kinases, control cellular phosphorylation levels [231]. Enzyme inactivation was facile with protein hydroperoxides and particularly with species generated on Tyr residues, as might perhaps be expected from the native substrates for these enzymes [232]. Inhibition was hydroperoxide dependent, and occurred with peroxide concentrations as low as 1 μM. These protein peroxides may therefore have major effects on cell signalling and processes dependent on protein phosphorylation.

The critical Ca^2+^ pump SERCA (sarco/endoplasmic reticulum Ca-ATPase) is also modified by amino acid- and peptide-hydroperoxides with a sub-set of the 22 reduced Cys residues present being modified, including Cys^674^ and Cys^675^ [233]. The first of these (in SERCA2) is a major target for NO-dependent *S*-glutathiolation of the protein, suggesting that hydroperoxide-mediated modification may be of significance in perturbing NO-dependent muscle relaxation [233]. It is well established that SERCA activity declines in aged tissues [234,235], a situation where protein hydroperoxides may be more prevalent.

The apparent commonality of the above chemistry suggests that other Cys-dependent enzymes will also be susceptible to amino acid and protein hydroperoxides. It also appears damage can occur at faster rates than with H_2_O_2_, suggesting that these species may be significant contributors to cellular redox changes.

### Modulation of protein turnover

Protein oxidation has been proposed as a marker for protein degradation by cellular machinery [236,237], with incomplete metabolism resulting in cellular accumulation of damaged, poorly degraded proteins. This has been associated with multiple human pathologies. The most well established are neurodegenerative pathologies (e.g. Alzheimer's disease, Parkinson's disease, Creutzfeldt–Jakob disease [238]), but accumulation of modified proteins with aging and disease appears to be a common characteristic [239–245]. Low levels of protein modification appear to result in enhanced turnover compared with native proteins, whereas extensive modification results in decreased turnover [246–256]. Turnover can occur via multiple cytosolic pathways (20S proteasome and lysosomal activity), in nuclei, and in mitochondria (Lon and Clp proteases) [256–259], with possible cross-talk between these systems [241,248,249]. Less is known about the turnover of modified extracellular proteins, though some endocytosis and lysosomal degradation may occur [260]. The slow turnover of large (and often heavily cross-linked) matrix proteins results in long half-lives [245,260], and this is likely to contribute to the accumulation of modifications on these materials [261–263]. Thus ∼70% of modifications detected in human atherosclerotic lesions have been reported to be present on matrix proteins [264].

Current data are consistent with most catabolism of modified proteins occurring via the (ATP-independent) 20S and immunoproteasomes [250], with metabolism of *native* proteins occurring via the ATP-dependent 26S form [249,257]. These systems can be regulated and inhibited by redox processes, with function declining with age and accelerated by disease [249,265–267]. Amino acid-, peptide- and protein-hydroperoxides can decrease the chymotryptic and tryptic (and also probably the caspase-like) activities of the 26S proteasome in both isolated systems and cell lysate preparations [268]. For purified human 26S proteasomes, inhibition appears to be associated, at least partly, with oxidation of a critical Cys on the S6 ATPase control subunits [268]. Inactivation occurs with low micromolar concentrations of peroxides, and at much lower concentrations than with H_2_O_2_ [268]. Hydroperoxide degradation products (probably aldehydes) also appear to play a role, as decomposed hydroperoxides also induced some inhibition [268]. 26S inhibition may modify the metabolism of proteins (e.g. NFκB and Nrf2) critical for cell signalling and oxidant response pathways, such that low levels of protein modification and hydroperoxide formation may have effects on normal cellular metabolism. As discussed above, amino acid and protein hydroperoxides are also efficient inhibitors of some lysosomal thiol-dependent cathepsins (B, L and S) [229], suggesting that hydroperoxides can inhibit most of the major catabolic pathways that are supposed to remove modified proteins, and this has led to the suggestion that this may constitute a vicious cycle in which protein hydroperoxide formation results in damage to the removal systems, which will then result in increased accumulation of modified/damaged proteins, an enhanced rate and extent of secondary damage, and a spiral of increasing inhibition and damage [229,268].

### Induction of DNA damage

Exposure of histone proteins to γ-irradiation in the presence of O_2_ results in high hydroperoxide concentrations (up to 70% of the initial radicals) [269,270], possibly as result of the high Lys and Arg content of these proteins–and the low levels of Cys, Met and aromatic residues that might act as alternative targets, or (in the case of Cys and Met) reducing agents for these species. Reaction of these peroxides with Cu^+^ and Fe^2+^ results in protein radicals (as detected by EPR spin trapping [269,270]), that react with pyrimidine bases and nucleosides to give protein–DNA base adducts [269–271], as well as the mutagenic product 7,8-dihydro-8-oxo-2′-deoxyguanosine (8-oxodG) [269,270]. The latter was also detected with intact DNA, and predominates in complex systems [272]. Strand-cleavage has been detected with plasmid DNA [273], and radio-labelling studies and gel-shift assays have suggested a significant role for hydroperoxides, and particularly lysine-derived species, in DNA-protein cross-linking reactions [271,273]. As histones are closely associated with DNA in the nucleus, and are known to bind copper ions that can catalyse hydroperoxide decomposition to radicals, these reactions may contribute to DNA–protein cross-links in damaged cells [269–271,273].

### Induction of lipid damage

Little work has been carried out to date on the induction of lipid oxidation by amino acid-, peptide- or protein-hydroperoxides, though it has been shown in some (but not all) cases that protein hydroperoxide formation may precede lipid oxidation. It is therefore possible that radicals formed from amino acid-, peptide- and protein-hydroperoxides may contribute to the initiation of lipid oxidation [182,185].

### Induction of secondary damage in cells

Evidence has been presented for changes in the redox status of cells on exposure to amino acid-, peptide- and protein- hydroperoxides. Photo-oxidation using the sensitizer Rose Bengal has been used to generate peroxides *in situ*, or prior to addition to cells, with the effects of the hydroperoxides examined after the cessation of illumination. In these systems a role for aldehydes generated from, or in parallel to, hydroperoxides cannot be completely excluded, though comparison of intact and reduced hydroperoxides, has allowed data to be obtained on this point. With murine macrophage-like (J774A.1) cells, pre-formed peptide hydroperoxides induced a loss of total cellular thiols and GSH that occurred concurrently with hydroperoxide consumption, and prior to a loss of cell viability [274]. GSH loss was more rapid than with the total thiol pool, suggesting that protein thiols are spared by GSH. This loss was not observed with decomposed hydroperoxides, and less marked effects were seen with large protein hydroperoxides. Inhibition of cathepsins B and L, and caspases 3/7 was detected, but not for other (non-thiol dependent) cathepsins or aryl sulfatase. These effects on lysosomal enzymes are consistent with hydroperoxide uptake and accumulation in the endo-lysosomal compartment [274].

When hydroperoxides were generated *in situ* in cells by limited photo-oxidation, a wide range of effects were detected that may arise from either direct reactions of transient intermediates (radicals or excited state) or peroxides. These effects included loss of GSH and total thiols, inhibition of multiple Cys-dependent (but not non-thiol) enzymes (e.g. GAPDH, thioredoxin, protein tyrosine phosphatases, creatine kinase, and cathepsins B and L), increases in NADPH levels and enhanced activity of gluthathione reductase, glutathione peroxidase and thioredoxin reductase. Each of these enzymes is associated with oxidative defences [187]. After cessation of illumination (i.e. during hydroperoxide decay) a limited initial recovery of thiol levels was detected, compared with the concentrations detected immediately after cessation of illumination, but these decreased with further incubation. Increases in the activity of GAPDH and protein tyrosine phosphatases were also detected, together with a marked increase in caspase 3/7 activity, and a loss of cell viability. These observations have been rationalized as limited repair and recovery, but ultimately the induction of apoptosis [187].

## CONCLUSIONS

The data reviewed above are consistent with proteins being major targets for biological oxidants due to their concentration, and high rate constants for reaction with multiple oxidants. In specific cases up to ∼70% of the initial oxidant results in hydroperoxide formation, and these values may be underestimated due to the instability of these species and problems in rapid and accurate quantification. High concentrations of hydroperoxides can also be generated by species such as ^1^O_2_. Existing data indicate that these species can be present at micromolar levels or higher in both intact cells and diseased tissues, and that total cumulative concentration of hydroperoxides to which proteins in diseased tissues have been exposed can be at least 400 μmol. These hydroperoxides can be long-lived in the absence of other reagents and are readily detected on isolated proteins, lipoproteins, in plasma, in cell lysates and some intact cells. Many of these hydroperoxides are poorly removed by well-established cellular protective systems that remove other oxidants such as H_2_O_2_, consistent with their much longer biological lifetimes compared with H_2_O_2_ and their detection in intact cells. These materials react at variable rates, depending on their structure and size, with one- and two-electron reducing agents. The one-electron reduction gives rise to additional radicals that contribute to protein chain oxidation reactions, cleavage of the protein back-bone, and modification of side chains via a complex series of hydrogen abstraction, electron transfer, fragmentation and O_2_ addition reactions. Some of these generate carbonyls and alcohols, well-established products of protein oxidation that accumulate in aged and some diseased tissues. Intermolecular reactions of hydroperoxide-derived radicals can result in DNA damage including the formation of 8-oxodG, protein–DNA base adducts and cross-links as well as strand cleavage. Two-electron reactions occur predominantly with Cys, selenium species, and to a lesser extent Met. These reactions are often more rapid than with H_2_O_2_ and can result in rapid and efficient inactivation of multiple cellular enzymes including those involved in calcium handling, phosphorylation, energy metabolism, apoptosis and protein turnover.

Although considerable data have been obtained on these species, there are still large gaps in our knowledge. Considerable additional work is needed to fully understand the role of these apparently common intermediates in protein modification and damage.

## Abbreviations

FOX: ferric iron–xylenol orange
GAPDH: glyceraldehyde-3-phosphate dehydrogenase
GPx: glutathione peroxidase
HOCl: hypochlorous acid
8-oxodG: 7,8-dihydro-8-oxo-2′-deoxyguanosine
PTP: protein tyrosine phosphatases
SERCA: sarco/endoplasmic reticulum Ca-ATPase
